# Impact of Antioxidants on Conventional and Advanced Sperm Function Parameters: An Updated Review

**DOI:** 10.7759/cureus.54253

**Published:** 2024-02-15

**Authors:** Mohannad Alharbi

**Affiliations:** 1 Department of Urology, College of Medicine, Qassim University, Qassim, SAU

**Keywords:** sperm dna, vitamins, oxidative stress, semen, male infertility, antioxidant

## Abstract

Antioxidants include diverse exogenous and endogenous compounds that can neutralize free radical activity, which ultimately protects sperm from oxidative stress (OS). Nevertheless, a controlled balance between oxidation and reduction is of paramount importance for cellular function. Excessive use of antioxidants should be avoided. A combination of antioxidants has been utilized to obtain a synergetic effect in the treatment of male infertility. Antioxidants have been shown to have a positive effect on semen parameters with a decrease in DNA damage. Future large randomized controlled trials are needed to determine the real impact of antioxidants on semen parameters, reproductive outcomes, and DNA integrity.

## Introduction and background

Antioxidants include diverse endogenous and exogenous compounds that can neutralize free radical activity and protect sperm from oxidative stress (OS). Exogenous antioxidants have two forms: dietary and oral supplements (natural extracts, herbs, and synthetic). The most frequently used compounds include vitamin E, vitamin C, carnitine, N-acetylcysteine (NAC), folic acid, lycopene, coenzyme Q10 (CoQ10), selenium, and zinc. There are risks and benefits to using these supplements. Antioxidants have been shown to decrease reactive oxygen species (ROS) and OS and protect against DNA damage. However, a controlled balance between oxidation and reduction is of paramount importance for cellular function. Excessive antioxidants can result in an “antioxidant paradox,” where a shift occurs from the redox balance to a more oxidized status, which leads to OS [1].

## Review

Effect of oral antioxidants on semen parameters

Several published studies have assessed the impact of various antioxidant treatments on semen parameters. The main limitations of the available evidence include small sample sizes, short follow-up, different antioxidant regimens with variable doses, and lack of proper control in the baseline demographic data. A recent Cochrane Collaboration that included 48 randomized controlled trials (RCTs) assessing the effect of antioxidants in subfertile men revealed a significant variability in the effect of antioxidants on sperm parameters [2].

Vitamin C

In 1992, Dawson et al. included 75 male smokers and observed that vitamin C supplementation with a daily dose of 200 mg or 1000 mg resulted in an improvement in sperm quality compared to the placebo group [3]. Another study explored the effect of vitamin C (500 mg daily) in 115 infertile men for three months following varicocelectomy and they showed an improvement in sperm motility and morphology, but there was no improvement in sperm count [4]. Similarly, Gual-Frau et al. included 20 infertile patients with asthenoteratozoospermia and clinical grade 1 varicocele to examine the effect of multivitamin supplementation containing vitamin C (60 mg) for three months and observed a statistically significant improvement in the total sperm number, but other sperm parameters were unchanged [5]. Moreover, the effect of a vitamin C-containing regimen (vitamin C 80 mg, vitamin E 40 mg, CoQ10 120 mg) was examined in 169 patients with oligoasthenozoospermia after three and six months of supplementation [6]. The authors reported an improvement in sperm concentration and motility. Omu et al. investigated the effect of administering a combination of zinc (200 mg), vitamin E (10 mg), and vitamin C (10 mg) in 45 asthenozoospermic men, and a statistically significant increase in sperm motility was noted [7]. Furthermore, Akmal et al. conducted a prospective study on infertile patients with oligozoospermia and reported an improvement in sperm motility, sperm count, and sperm morphology after two months of vitamin C supplementation (2000 mg daily) [8]. 

Many investigators have found that vitamin C supplementation improved semen parameters. However, other studies failed to show such findings. Rolf et al. conducted a prospective randomized placebo-controlled double-blind trial of 31 infertile men with asthenozoospermia [9]. Patients were randomized to receive either 1000 mg vitamin C and 800 mg vitamin E or placebo pills for 56 days. The trial showed no improvement in semen parameters after supplementation compared to placebo. Similarly, Greco et al. evaluated the effect of two months of treatment with 1 g vitamin C and 1 g vitamin E daily in men with idiopathic infertility and found that antioxidants had no effect on sperm parameters compared to the placebo group [10]. 

Vitamin E

A study by Suleiman et al. (1996) showed that 87 infertile patients with asthenozoospermia were included and randomized to vitamin E (300 mg daily) or placebo for six months [11]. The authors reported an improvement in sperm motility. Subsequent studies examined the combination of vitamin E with other minerals and vitamins. ElSheik et al. performed a prospective randomized trial enrolling 90 infertile men and reported that the combination of vitamin E (400 mg daily) and clomiphene citrate (25 mg daily) for six months resulted in an improvement in sperm motility and concentration [12]. In another prospective study, the combination of vitamin E (400 units) and selenium (200 μg) for 100 days led to a 52.6% improvement in sperm motility and morphology [13]. 

However, two other randomized double-blind placebo-controlled studies showed no improvement in semen parameters after vitamin E ingestion. Moilanen et al. reported that treatment with vitamin E (300 mg daily) for three months did not reveal any significant improvement in sperm concentration, motility, and morphology [14]. Additionally, Kessopoulou et al. conducted a study that enrolled 30 infertile men with high ROS and showed that there was no improvement in the semen parameters after vitamin E (600 mg daily) supplementation for three months [15].

Carnitines 

One of the earliest reports was published by Lenzi et al. when they performed a double-blind cross-over trial and they included 100 infertile men to assess the effect of L-carnitine (LC) (2 g daily) and they showed an improvement in sperm concentration and motility [16]. The improvement in motility was more pronounced in men with a lower baseline sperm motility. One year later, the same group published their experience using LC (2 g daily) combined with L-acetyl-carnitine (LAC) (1 g daily), an improvement in all sperm parameters was noted with a similar trend between motility and low initial values [17]. Similarly, Balercia et al. examined the effect of an LC and LAC combination and found a significant increase in sperm motility and total oxygen radical scavenging capacity [18]. Moreover, a link was found between the physiologic function of mitochondria, which is reflected by phospholipid hydroperoxide glutathione peroxidase (PHGPx) and carnitine supplementation [19]. Garolla et al. observed that carnitine treatment improved sperm motility in infertile men with a normal PHGPx level [19]. Additionally, a combination of LC (1 g), LAC (500 mg), fumarate (725 mg), fructose (1 g), CoQ10 (20 mg), vitamin C (90 mg), zinc (10 mg), folic acid (200 μg), and vitamin B12 (1.5 μg) was examined in patients with oligoasthenoteratozoospermia (OAT) with and without varicocele [20]. The authors found a significant increase in sperm concentration and motility compared to placebo and the effect was more apparent in the varicocele group [20].

However, a prospective randomized double-blind placebo-controlled trial by Sigman et al. failed to show any significant effect on sperm motility after LC and LAC supplementation in men with idiopathic asthenozoospermia [21].

Coenzyme Q10 

CoQ10 was assessed in a randomized double-blind placebo-controlled trial where 60 men with idiopathic infertility were randomized to receive CoQ10 (200 mg daily) or placebo for six months [22]. The result revealed a statistically significant increase in motility especially in patients with a lower initial motility value [22]. Another study examined the effect of CoQ10 (300 mg daily) in 212 infertile men compared to placebo and they showed an improvement in sperm concentration and motility after 26 weeks of supplementation [23]. Nadjarzadeh et al. investigated the effect of CoQ10 on OS and antioxidant enzymes in the semen and they observed an improvement in catalase and superoxide dismutase activities that were associated with an increase in sperm morphology [24]. Similarly, Thakur et al. showed that daily supplementation of 150 mg CoQ10 significantly improved all sperm parameters in infertile patients [25]. However, a double-blind placebo-controlled randomized trial was performed and included 47 men with idiopathic OAT. The patients were randomized to receive CoQ10 (200 mg daily) or placebo for 12 weeks, and they found an increase in total antioxidant capacity with a decrease in lipid peroxidation but with no effect on semen parameters [26].

N-acetylcysteine

NAC (600 mg daily) was shown to improve semen volume, motility, and viscosity in 120 men with idiopathic infertility compared to placebo [27]. Moreover, Safarinejad et al. explored the effect (600 mg NAC + 200 μg selenium) on 468 patients with idiopathic OAT for 26 weeks [28]. The authors found an improvement in all sperm parameters and the combination had an additive effect [28]. 

Selenium

Selenium has been examined in combination with either vitamin E or NAC. The vitamin E (400 mg) and selenium (225 μg) combination for three months produced an improvement in sperm motility and a decrease in malondialdehyde (MDA), which is a marker for lipid peroxidation [29]. Another clinical trial was performed by Moslemi et al. where the combination of vitamin E (400 units) and selenium (200 μg) for 100 days resulted in a 52.6% improvement in sperm motility and morphology [13]. As mentioned in the NAC section, a combination of 600 mg NAC + 200 μg selenium for 26 weeks improved all semen parameters. However, Hawkes et al. showed no improvement in semen parameters after 48 weeks of selenium supplementation (300 μg daily) in healthy normozoospermic men [30].

Zinc

A study by Omu et al. (1998) examined the effect of zinc (500 mg daily) for three months in 100 patients with astenozoospermia and randomized them to receive zinc or no treatment [31]. The study showed an improvement in sperm concentration and motility with a decline in antisperm antibodies [31]. Hadwan et al. published two studies in 2014 and 2015, which included infertile men with asthenozoospermia and compared them to a fertile group. They were given zinc (440 mg daily) for three months and the results showed an improvement in sperm concentration and motility. They also observed that catalase-like activity, peroxynitrite levels, nitric oxide synthase activity, and arginase activity were restored to normal ranges in infertile men [32,33]. Moreover, zinc has been examined in combination with either vitamin E and vitamin C or folic acid. In 2008, Omu et al. assessed the effect of administering a combination of zinc (200 mg), vitamin E (10 mg), and vitamin C (10 mg) in 45 asthenozoospermic men, and they reported a statistically significant increase in sperm motility [7]. Additionally, the combination of zinc (66 mg) and folic acid (5 mg) for 26 weeks in fertile and infertile men resulted in a 74% increase in total sperm count in the infertile group [33]. A similar regimen was used in another study with a similar duration and it showed an improvement in sperm concentration only, with no effect on other semen parameters [33]. 

Folic acid

As previously shown in the zinc section, two studies by Wong et al. and Ebisch et al. have revealed the beneficial effect of combining zinc and folic acid on total sperm count and concentration [34,35]. However, Raigani et al. did not show any improvement in sperm concentration following zinc (220 mg daily) and folic acid (5 mg daily) treatment for 16 weeks in 83 men with OAT [36].

Lycopene

Lycopene was shown to significantly improve sperm concentration and motility in two reports that included patients with idiopathic OAT [37,38].

Combination of oral antioxidants

The theory behind the current practice of using combination antioxidants is to obtain a synergetic effect in the treatment of male infertility. One of the earliest studies was performed by Comhaire et al. (2000) and they found that the combination of NAC or vitamins A and E and essential fatty acids resulted in a decrease in ROS activity with an increase in sperm concentration, but no improvement in sperm motility or morphology [39]. Similarly, another study included men with persistent oligozoospermia six months after varicocele embolization. The patients were given NAC, vitamin C, vitamin E, vitamin A, thiamine, riboflavin, biotin, B12, zinc, and other minerals. Results showed an improvement in sperm count with no effect on sperm motility and morphology [40]. Piomboni et al. conducted a study on 36 infertile men with asthenoteratozoospermia and leukocytospermia and noticed a significant improvement in motility and morphology with a decrease in leukocytospermia after 90 days of antioxidant supplementation (beta-glucan, papaya, lactoferrin, 60 mg vitamin C, and 10 mg vitamin E) [41]. Similarly, Wirleitner et al. observed an improvement in all semen parameters after an antioxidant combination (200 mg vitamin C, 200 mg vitamin E, 1 mg folic acid, 50 mg zinc, 200 μg selenium, 100 mg NAC, and 600 mg LC) for at least two months [42]. Similar results were found by using the same regimen but at a lower dose in other studies [43,44]. 

However, other studies did not show an improvement in semen parameters after combination antioxidant supplementation [9,10,34]. The Cochrane review that included 48 RCTs examining the effect of antioxidants in subfertile men showed significant variability in the effect of antioxidants on sperm parameters [2]. More recently, Stenqvist et al. conducted a double-blind placebo-controlled randomized trial that included 77 infertile men with a high DNA fragmentation index (DFI) >25% [45]. Subjects were randomized to antioxidant treatment twice per day (30 mg vitamin C, 5 mg vitamin E, 100 μg folic acid, 5 mg zinc, 25 μg selenium, 0.5 μg vitamin B12, 750 mg LC, and 10 mg CoQ10) or placebo for six months. The authors showed no statistically significant improvement in any of the semen parameters after six months [45]. 

Effect of antioxidants on oxidative stress and DNA damage

The fertilization process and subsequent embryo development depend partially on the inherent integrity of the sperm DNA. There may be a threshold of sperm DNA damage (i.e. sperm DNA fragmentation (SDF), abnormal chromatin packaging, and protamine deficiency) beyond which fertilization and embryo development are impaired [46]. Several studies have investigated the effect of antioxidants on sperm DNA integrity [47,48,49].

One of the first reports that examined the association between antioxidants and sperm DNA was published by Fraga et al. (1991) when they assessed the effect of vitamin C repletion in 10 subjects who were maintained on a controlled intake of vitamin C for 15 weeks [47]. The study showed a significant decrease of 36% in 8-hydroxy-2ʹ-deoxyguanosine (8-OHdG), which is a measure of oxidative DNA damage [47]. Similarly, Kodama et al. found a significant reduction in 8-OHdG in 14 infertile men after two months of antioxidant supplementation (200 mg vitamin C, 200 mg vitamin E, and 400 mg glutathione) [48]. In another study by Greco et al., the combination of 1 g vitamin C and vitamin E daily for two months in men with idiopathic infertility and high SDF resulted in a significant decrease in SDF compared to the placebo group [10]. They used terminal deoxyribonucleotidyl transferase-mediated dUTP nick-end labeling (TUNEL) to assess SDF. Moreover, a prospective study showed a decrease in SDF (measured by sperm chromatin structure assay (SCSA)) but there was an unexpected increase in %high DNA stainability (%HDS) after three months of vitamin C and E (400 mg), zinc (500 mmol), selenium (1 mmol), and b-carotene supplementation [49]. Tunc et al. examined the effect of Menevit (100 mg vitamin C, 400 IU vitamin E, 25 mg zinc, 26 mg selenium, 0.5 mg folic acid, lycopene, and garlic oil) in 45 infertile men. The authors revealed a significant decrease in SDF (measured using TUNEL), an increase in sperm protamination, and a decrease in ROS production [50]. Omu et al. also assessed the effect of administering the combination of zinc (200 mg), vitamin E (10 mg), and vitamin C (10 mg) in 45 asthenozoospermic men, and they found a statistically significant decrease in SDF (measured using SCSA) [7]. Two other prospective studies that used the sperm chromatin dispersion test (SCD) reported a significant decrease in SDF and an improvement in DNA-degraded sperm (DDS) after antioxidant supplementation [5,44].

Conversely, other studies failed to show an effect of high SDF by antioxidants. Silver et al. (2005) included 87 healthy men to study the effect of antioxidant intake (vitamin C, vitamin, E, and b-carotene) on sperm chromatin integrity, and they observed that no association exists between antioxidants and SDF (measured using SCSA) and HDS. Only subjects with moderate b-carotene intake had high SDF and HDS as a side effect [34]. Piomboni et al. also performed a study on 36 infertile men with asthenoteratozoospermia and leukocytospermia and showed no significant decrease in SDF (measured with SCSA) after 90 days of antioxidants supplementation (beta-glucan, papaya, lactoferrin, 60 mg vitamin C, and 10 mg vitamin E) [41]. Furthermore, Raigani et al. conducted a double-blind randomized trial in 83 men with OAT who received zinc (220 mg daily) and folic acid (5 mg daily) supplementation for 16 weeks [34]. The study showed a trend toward a decrease in SDF (measured using SCSA), but a statistically significant increase in aniline blue staining (AB). Recently, Stenqvist et al. conducted a double-blind placebo-controlled randomized trial that included 77 infertile men with a high DFI >25% [45]. Participants were randomized to receive antioxidant treatment twice per day (30 mg vitamin C, 5 mg vitamin E, 100 μg folic acid, 5 mg zinc, 25 μg selenium, 0.5 μg vitamin B12, 750 mg LC, and 10 mg CoQ10) or placebo for six months. The authors showed no statistically significant change in SDF (measured using SCSA) after six months [45].

Several investigators have assessed the effect of antioxidants on seminal OS activity [45,46,47]. Various antioxidants led to a reduction in MDA, which is a marker for lipid peroxidation [7,11,27,29,49]. Additionally, antioxidants have been found to decrease seminal ROS levels in many reports [39,50,51,52]. 

Effects of antioxidants on reproductive outcomes

The beneficial effects of antioxidants on semen parameters and sperm quality have been established in many studies, but their effect in pregnancy is not well-defined (Table 1) [1]. Kobori et al. reported a pregnancy rate of 28% in 169 patients with oligoasthenozoospermia after three and six months of supplementation (vitamin C 80 mg, vitamin E 40 mg, and CoQ10 120 mg) [6]. Similarly, vitamin E supplementation resulted in a pregnancy rate of 11% and 21% in two studies [11,13]. Moreover, individual supplementation of zinc and carnitine was found to improve the pregnancy rate by over 13% [17,31]. Furthermore, a combination of LC (1 g), LAC (500 mg), fumarate (725 mg), fructose (1 g), CoQ10 (20 mg), vitamin C (90 mg), zinc (10 mg), folic acid (200 μg), and vitamin B12 (1.5 μg) was assessed in patients with OAT with and without varicocele (20). Although the pregnancy rate was not an endpoint in the study, the authors found 10 pregnancies in the treatment group compared to two pregnancies in the placebo group [20]. However, Rolf et al. reported that vitamin C and E supplementation did not result in pregnancy in a randomized placebo-controlled study, as follows: 0% (0/15) vs. 0% (0/16) [9]. Although antioxidant supplementation resulted in an improved pregnancy rate in two other studies, the difference between treatment and untreated/placebo groups was not statistically significant [40,53]. The Cochrane collaboration included 48 RCTs that assessed the effect of antioxidants in subfertile men, which showed a statistically significant increase in the live birth rate and clinical pregnancy rate [2].

**Table 1:** Summary of clinical trials (RCTs) examining the effect of antioxidants on pregnancy rates. IUI, intrauterine insemination; CPR, clinical pregnancy rate; ICSI, intracytoplasmic sperm injection; DFI, DNA fragmentation index; NAC, N-acetylcysteine; LC, L-carnitine; LAC, L-acetyl-carnitine; OAT, oligoasthenoteratozoospermia; CoQ10, Coenzyme Q10

Study	Population	Antioxidant	Results
Attallah et al. (2013) [59]	Couples with isolated idiopathic athenozospermia prior to IUI	NAC	20.0% CPR in the antioxidant group compared with 13.3% CPR in the control group (P=0.25).
Balercia et al. (2005) [18]	Infertile men with idiopathic asthenozoospermia	LC and LAC	Nine pregnancies in the antioxidant group and three pregnancies in the placebo group
Balercia et al. (2009) [22]	Infertile men with idiopathic asthenozoospermia	CoQ10	Six pregnancies in the antioxidant group and three pregnancies in the placebo group
Busetto et al. (2018) [20]	OAT with and without varicocele	LC, LAC, fumarate, fructose, CoenzymeQ10, vitamin C, zinc, folic acid, and vitamin B12	Ten pregnancies in the antioxidant group (one varicocele, nine in non-varicocele) and two pregnancies in the placebo group
Galatioto et al. (2008) [40]	Men with persistent oligozoospermia 6 months after varicocele embolization	NAC, vitamin C, vitamin E, vitamin A, thiamine, riboflavin, biotin, vitamin B12, zinc	No impact on spontaneous pregnancies in antioxidant group vs. control group (5% vs. 0%, P = 0.95)
Lenzi et al. (2003) [16]	Infertile patients with OAT	LC	Six pregnancies in the antioxidant group and zero pregnancy in the placebo group
Lenzi et al. (2004) [17]	Infertile patients with OAT	LC and LAC	Four pregnancies in the antioxidant group and zero pregnancy in the placebo group
Sigman et al. (2006) [21]	Infertile men with idiopathic asthenozoospermia	LC and LAC	No effect on pregnancy rate
Tremellen et al. (2007) [56]	Infertile men before their partner’s ICSI cycle	Menevit formulation	Significant improvement in viable pregnancy rate in couples during ICSI compared to the control group (38.5% vs. 16%, P = 0.046)
Stenqvist et al. (2018) [45]	Infertile men with a high DFI > 25%	Vitamin C, vitamin E, folic acid, zinc, selenium, vitamin B12, LC, and CoQ10	Three pregnancies in the antioxidant group and four pregnancies in the placebo group

The effect of antioxidants on assisted reproductive technology (ART) was examined in a few studies. Vitamin E was found to improve in vitro fertilization (IVF) rates and sperm zona pellucida binding [11,15,54]. Additionally, Greco et al. evaluated the effect of two months of treatment with 1 g vitamin C and vitamin E daily on the second intracytoplasmic sperm injection (ICSI) attempt in men with high SDF and who had a failed first ICSI attempt [55]. The study found no difference in cleavage and fertilization rates, but a significant increase in the clinical pregnancy rate (6.9% to 48.2%), and the implantation rate (2.2 to 19.6%) was noted [55]. Tremellen et al. conducted a randomized placebo-controlled double-blind trial comparing the Menevit formulation with placebo in infertile men before their partner’s ICSI cycle [56]. They reported a significant improvement in the clinical pregnancy rate compared to the placebo group but with no improvement in the fertilization rate or embryo quality between the two groups [56]. Finally, CoQ10 resulted in an increase in the fertilization rate in ICSI in subjects with a low fertilization rate in a previous ICSI cycle (10.3% vs. 26.3%, P < 0.05) [57,58].

Adverse effects of antioxidants: “antioxidant paradox”

Excessive antioxidants can rarely result in an “antioxidant paradox,” where a shift occurs from the redox balance to a more oxidized status, which leads to OS [58]. The “antioxidant paradox” has a detrimental effect at the cellular level, which is similar to the effect seen with OS. There is some emerging evidence that antioxidants if used excessively can result in a paradoxical decline in sperm quality. However, an exact cut-off for "excessive" has not been well defined in the existing literature [58,59].

## Conclusions

Antioxidants have been shown to have a positive effect on semen parameters with a decrease in DNA damage. However, the available studies included small sample sizes, short follow-ups, different antioxidant regimens with variable doses, and lack of a proper control in the baseline demographic data. Future large RCTs are needed to determine the real impact of antioxidants on semen parameters, reproductive outcomes, and DNA integrity.
